# Effect of Extra Virgin Olive Oil and Traditional Brazilian Diet on the Bone Health Parameters of Severely Obese Adults: A Randomized Controlled Trial

**DOI:** 10.3390/nu12020403

**Published:** 2020-02-04

**Authors:** Camila Kellen de Souza Cardoso, Annelisa Silva e Alves de Carvalho Santos, Lorena Pereira de Souza Rosa, Carolina Rodrigues Mendonça, Priscila Valverde de Oliveira Vitorino, Maria do Rosário Gondim Peixoto, Érika Aparecida Silveira

**Affiliations:** 1 The Postgraduate Program in Health Sciences, School of Social Sciences and Health, Nutrition Course, Pontifical Catholic University of Goiás, Goiânia, 74605-020 Goiás, Brazil; camilacardoso_nut@hotmail.com; 2 The Postgraduate Program in Health Sciences, Faculty of Medicine, Federal University of Goiás, Goiânia, 74605-220 Goiás, Brazil; annelisa.nut@gmail.com (A.S.e.A.d.C.S.); lorenapsrosa@yahoo.com.br (L.P.d.S.R.); carol_mendonca85@hotmail.com (C.R.M.); 3 The Postgraduate Program in Health Sciences, Professor of the School of Social Sciences and Health, Pontifical Catholic University of Goiás, Goiânia, 74605-020 Goiás, Brazil; pvalverde@pucgoias.edu.br; 4 The Postgraduate Program Nutrition and Health, Faculty of Nutrition, Federal University of Goiás, Goiânia, 74605-220 Goiás, Brazil; mrg.peixoto@uol.com.br

**Keywords:** bone mineral density, obesity, healthy diet, parathyroid hormone, calcium and vitamin D, clinical trial

## Abstract

Dietary interventions can stabilize and/or reverse bone mass loss. However, there are no reports on its effects on bone mineral density (BMD) in severely obese people, despite the vulnerability of this group to bone loss. We examine the effect of extra virgin olive oil supplementation and the traditional Brazilian diet (DieTBra) on BMD and levels of calcium, vitamin D, and parathyroid hormone (PTH) in severely obese adults. A randomized controlled trial followed-up with severely obese adults (*n* = 111, with mean body mass index 43.6 kg/m^2^ ± 4.5 kg/m^2^) for 12 weeks. Study participants received either olive oil (52 mL/day), DieTBra, or olive oil + DieTBra (52 mL/day + DieTBra). BMD was assessed by total spine and hip dual-energy X-ray absorptiometry. After interventions, BMD means for total spine (*p* = 0.016) and total hip (*p* = 0.029) were higher in the DieTBra group than in the olive oil + DieTBra group. Final mean calcium levels were higher in the olive oil group compared to the olive oil + DieTBra group (*p* = 0.026). Findings suggest that DieTBra and extra virgin olive oil have positive effects on bone health in severely obese adults. The major study was registered at ClinicalTrials.gov (NCT02463435).

## 1. Introduction

Severe obesity, defined by a body mass index (BMI) ≥35 kg/m^2^, a category of obesity that has majorly increased in the last years, is an important risk factor for increased morbidity and mortality, associated with the increased occurrence of chronic non-communicable diseases [1,2]. Severe obesity appears to not have a protective effect on bone health [3], although the research is still incipient and controversial [4,5,6]. Increased prevalence of both severe obesity [1] and osteoporosis [7] is a noteworthy health concern.

Chronic state of inflammation, endocrine, and metabolic disorders common in obese adults act negatively on bone mass. These conditions may support osteoclast hyperactivity, followed by osteoblast hypoactivity, resulting in bone deterioration [5,6,8,9]. Dietary interventions to stabilize and/or reverse bone mass loss, such as the use of extra virgin olive oil, were examined [10,11,12,13,14,15].

Recent studies showed that women with a higher adherence to the Mediterranean diet (MD), which is also characterized by large amounts of olive oil intake, have higher bone mineral density (BMD) compared to those with lower adherence [16,17]. Extra virgin olive oil (EVOO) is rich in mono-unsaturated fatty acids (MUFAs), polyphenols, hydrocarbons, phytosterols, and tocopherols, which have anti-inflammatory and antioxidant properties [18]. These may increase the expression of type I collagen gene, the activity of alkaline phosphatase (ALP), fibronectin, the deposit of extracellular calcium ions, in addition to improving inflammation levels and benefiting osteoclast hypoactivity, resulting in increased bone mass [8,13,19,20,21,22].

Despite the benefits of healthy dietary patterns to bone health and obesity, no randomized controlled trials (RCTs) have evaluated the effect of dietary interventions on BMD and other parameters that affect bone health in severely obese adults. Including EVOO and a healthy dietary pattern into one’s diet can be positive to BMD and other metabolic parameters. The objective of this study is to analyze the effectivity of a nutritional intervention with EVOO and a healthy diet pattern on BMD, levels of serum calcium, vitamin D, and parathyroid hormone (PTH) in severely obese adults. The healthy diet pattern used in this study is the Brazilian traditional diet (DieTBra), characterized by the consumption of a variety of healthy foods including fruits, vegetables, and small amounts of meat [23], according to the Brazilian food culture before nutritional transition [24].

## 2. Methods

### 2.1. Design and Recruitment of Participants

This was a parallel RCT assessing a nutritional intervention (DieTBra) and supplementation with extra virgin olive oil in severely obese adults. The study was conducted at the Clinical Research Unit of the Clinics Hospital of the Federal University of Goiás (Goiânia, Goiás, Brazil), between June 2015 and February 2016. This study is part of the major RCT “Effect of nutritional intervention and olive oil in severe obesity—DieTBra Trial” [25,26,27,28]; details of the study design and subject recruitment and randomization were previously described [25,26,27,28].

**Figure 1:**
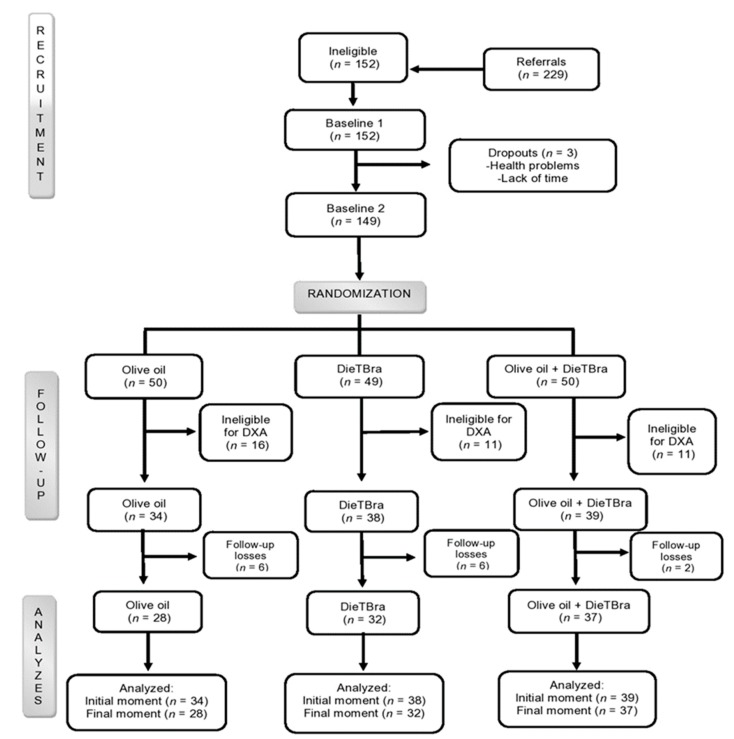
Tradition Brazilian diet (DieTBra) and extra virgin olive oil. DXA = dual-energy X-ray absorptiometry.

During data collection 229 participants went to the Nutrition and Severe Obesity Outpatient Clinic and 149 participants were enrolled in the study; of these, 111 participants were eligible for dual-energy X-ray absorptiometry (DXA) (Figure 1). Eligibility criteria were adults between 18 and 64 years [29], with BMI ≥35 kg/m^2^ [1], and body weight ≤130 kg (capacity of the bone densitometer) (Figure 1). The following exclusion criteria were applied: history of bariatric surgery; weight loss of >8% in the preceding 3 months; history of nutritional treatment for weight loss in the past two years; pregnancy; participants with previous or current cancer diagnosis; nursing mothers; physical or mental disabilities; intolerance to oils; and presence of metal in the body (rods and pins). These measures were adopted during the baseline and follow-up testing of the study participants.

### 2.2. Sample Calculation, Randomization, and Blinding

The sample size calculation was performed a posteriori [30], with a significance level (α) of 5% and a statistical power of 80%, a mean expected difference in BMD between treatments of 0.07 g/cm^2^, and a standard deviation of the mean difference of 0.092 g/cm^2^ for total spine BMD and 0.100 g/cm^2^ for total hip BMD. The final calculation resulted in 27 participants in each group. As this study is included in a multidisciplinary RCT, the DieTBra Trial, the total number of adults analyzed is superior to this sample size calculation.

Randomization was performed in a random allocation sequence with 1:1:1 ratio, using previously described procedures [25,26,27]. Thus, 149 participants were randomized into three groups: olive oil, traditional Brazilian diet (DieTBra), and olive oil + DieTBra (olive oil + DieTBra) (Figure 1).

It is almost impossible for nutritional interventions to be completely blind [31], although we applied some measures to prevent information exchange between groups. The three intervention groups had no contact with each other and attended the participant clinic on different days. During the data collection period, the research team replaced the word “extra virgin olive oil” for “dietary supplement”. Therefore, participants did not specifically know which food supplement they were receiving.

The dietary intervention was prescribed individually for the DieTBra group and the olive oil + DieTBra group. The DieTBra is characterized by the traditional Brazilian food pattern [32], defined by the consumption of non-processed foods in at least four meals/day. The main meals, lunch and dinner, include the consumption of rice, beans, lean meat, and vegetables. Throughout the day seasonal fruits are recommended in small meals. In breakfast and/or afternoon snack, dairy products, coffee, and homemade treats should be consumed. The DieTBra contains an adequate supply of vitamins, minerals, and dietary fiber with low content of trans and hydrogenated fats, consistent with a healthy eating pattern. The intervention with DieTBra is based on the attempt to rescue the Brazilian healthy food culture, common in the country before the nutritional transition process [33]. DieTBra fits into healthy and sustainable dietary patterns proposed by the World Health Organization [34], as an affordable diet for the general population not only in Brazil but also in other countries, with widely available foods in various regions of the world [23,34].

To determine the total energy value (TEV) of the DieTBra for each participant, the resting energy expenditure (REE) is calculated by a specific formula developed for obese people [35], using the fat-free mass index. The total energy expenditure (TEE) was calculated [36] including data on the level of physical activity according to the Global Physical Activity Questionnaire or GPAQ [37]. The physical activity factor is adapted from the Institute of Medicine [38] and Bouchard et al. [39], considering an 8% thermic effect of food (TEF) of the TEE [38,40].

To calculate the DieTBra we adopted a weight loss target of 5% to 10% from the initial weight, according to the BMI range: a reduction of 5%–6% for BMI ≥35 and <40 kg/m^2^; 7%–8% for BMI ≥40 and ≤50 kg/m^2^; and 9%–10% for BMI > 0 kg/m^2^. The Dietary Reference Intakes (DRIs) from the Institute of Medicine [38] are used to determine the distribution range of all macronutrients: carbohydrates, protein, and lipids.

Two intervention groups received extra virgin olive oil: the olive oil group and the olive oil + DieTBra group. We used extra virgin olive oil, cold pressed with <2% acidity, packaged in laminated sachets with a capacity of 13 mL, to facilitate the consumption of the supplement and to avoid portion size errors. At the end of each consultation, a quantity of extra virgin olive oil sufficient for four weeks was delivered. Study participants were instructed to consume two sachets at lunch and two sachets at dinner, totaling 52 mL/day. At each return visit, all sachets were collected and recorded as a quality control measure and the intake of extra virgin olive oil was evaluated.

The amount of extra virgin olive oil used in our study was about 42% higher than the standard portion reported in the literature (≤30 mL/day), considering losses during consumption [41,42]. The prescribed quantity of extra virgin olive oil accounted for 468 kcal/day. The extra energy value from olive oil was taken into consideration in the calculation of the diet TEV. Thus, the olive oil + DieTBra group received a high-fat diet (around 45% of the TEV), while in the DieTBra group, total lipid content was around 30% of the TEV. All intervention groups returned every four weeks, with a total follow-up time of 12 weeks.

### 2.3. Sociodemographic, Lifestyle, and Medical Conditions

We obtained data through a structured, standardized, and previously tested questionnaire. The sociodemographic variables were sex, age, skin color, formal education (total years of study), and economic class (Brazilian Association of Population Studies or ABEP). Smoking was classified into three categories, according to the Pan American Health Organization (PAHO) [43]: non-smoker, ex-smoker, and smoker. Excessive alcohol consumption (binge drinking) was categorized when the intake was ≥5 alcoholic drinks for men and ≥4 drinks for women on a single occasion [44]. Physical activity (PA) was evaluated using the “triaxial accelerometer ActiGraph wGT3X” (ActiGraph, Pensacola, FL, USA) and ActiLife 6 software positioned at the back of the non-dominant wrist. Each individual was instructed to use it 24 h a day for six consecutive days. The level of PA was categorized according to the recommended practice of ≥150 minutes/week of moderate to vigorous aerobic physical activity (MVPA) [45]. The outcome measures used in the present study were moderate-to-vigorous physical activity (MVPA) (>100 mg) defined as estimated time spent as ≥10 min per bout during the week [46].

The previous diagnosis of menopause was verified by asking the question: “The doctor has already said you are in menopause?”. The use of drugs that interfere with BMD was recorded by consulting the medical prescription and/or packaging and included: glucocorticoids, proton pump inhibitors, anticonvulsants/neuroleptics, medroxyprogesterone acetate (MPA), aromatase inhibitors, gonadotropin-releasing hormone agonists (GnRH), thiazolidinediones/glitazones, calcineurin inhibitors, heparin and warfarin/anticoagulant, thyroxine/thyroid hormone, loop diuretics, and calcium and vitamin D supplements [47,48].

### 2.4. Biochemical Tests, Anthropometry, and Dietary Intake

Blood tests were collected after 12 h of fasting. Serum calcium was evaluated by the endpoint colorimetric technique, Arsenaso III, while vitamin D (vitamin D_3_) and PTH were measured by the electrochemiluminescence method.

To measure current weight, we used a digital scale, platform type, with a capacity of 200 kg and accuracy of 100 g (Welmy, Santa Bárbara d’Oeste, SP, Brazil). Height was measured by a stadiometer coupled to the digital scale with a precision of 0.1 cm. Anthropometric measurements were collected according to standardized procedures [49]. The data on weight and height were used to calculate BMI [50].

The dietary intake variables—energy (kcal/day), protein (% total energy value), calcium (mg/day) and vitamin D (µcg/day)—were evaluated by the average of two 24-h recalls [51]. The pharmacist from the research group evaluated vitamins, supplements, and use of medications, and none of the participants reported the use of those products during the follow-up.

### 2.5. Bone Mineral Density and Body Composition

Spine (cervical, thoracic, and lumbar) and hip BMD were measured by dual-energy X-ray absorptiometry (DXA) using the GE Healthcare (Lunar DPX NT, Auckland, New Zealand) bone densitometer with a capacity of 130 kg and a width of 1.03 m. This exam was always performed by the same technician from the Clinical and Sports Nutrition Research Laboratory of the Faculty of Nutrition (LABINCE-FANUT) of the Federal University of Goiás, in the presence of one of the authors. At the time of the examination, the participant was positioned in dorsal decubitus with arms along the body, within the line allowed for the exam. If one of the participant’s arms remained out of the allowed line, the left arm was excluded from the analysis with automatic doubling of the value of the right arm by the equipment [52]. BMD was measured in g/cm^2^. In this whole-body scan, the total body fat (kg) was also evaluated, as well as the lean mass (kg) of the participants.

### 2.6. Statistical Analysis

The database was built using EpiData version 3.1, with double entry typing for further analysis of consistency and validation of information. Statistical analysis was performed using Stata/SE version 12.0 software.

The primary outcomes were the total spine BMD and total hip BMD. The secondary outcomes were the levels of serum calcium, vitamin D and PTH, dietary calcium and vitamin D, and energy and protein intake. All outcomes were evaluated continuously in order to determine changes at the end of the follow-up. We also calculated delta, namely, the variation of the outcomes between the beginning and end of the study. The Kolmogorov–Smirnov test was used to test the normality of the continuous variables.

In the descriptive analysis we calculated the absolute and relative frequencies, means, and standard deviation. An unpaired Student’s t-test was applied to evaluate the means within each group by intention-to-treat. The difference between the initial and final means was evaluated by Student’s t-test and analysis of variance (ANOVA) followed by Bonferroni or Mann–Whitney and Kruskal–Wallis tests for non-parametric data. Analysis of covariance (ANCOVA) was used with the following: outcome with a normal distribution; linear relationship between the outcome variable and covariate (delta weight); and homoscedasticity of variance [53]. Statistical significance was set at a *p*-value < 0.05.

The presence of outliers was evaluated by boxplot graphs for all the continuous variables. For the outcomes where the presence of outliers was identified, the analysis was performed with and without the outliers. If an influential outlier was identified, the mean or median value was assigned to it, which occurred only for the initial PTH variable.

### 2.7. Quality Control

All team members underwent training and standardization on the protocols of care, anthropometry, examination checklists, adherence approaches, and service routines. At the end of each consultation, the questionnaires were coded and checked by different team members. Data were entered in duplicate for the subsequent analysis of consistency and for quality assurance (validation). In addition, 36 standard operating procedures (SOPs) were developed to avoid errors and bias in the major study.

### 2.8. Ethical Aspects and RCT Registration

The DieTBra Trial was approved by the Research Ethics Committee of the Clinics Hospital of the Federal University of Goiás under protocol number 747.792/2014. All participants signed a written consent form. The major study was registered at ClinicalTrials.gov platform (NCT02463435).

## 3. Results

Baseline characteristics are shown in Table 1. Of the 111 severely obese participants in this clinical trial, 93.7% were women, with a mean age 40.5 ± 8.6 years (between 21 and 62 years) and a mean BMI of 43.6 kg/m^2^ ± 4.5 kg/m^2^ (between 35.0 and 54.8 kg/m^2^); the most frequent BMI category was from 40 to 49.9 kg/m^2^ (70.3%). Further, 97 participants (87.4%) had DXA scans performed at the beginning and end of follow-up (Figure 1). There was no difference between groups for the explanatory variables and outcomes at the beginning of the study (*p* > 0.05).

Regarding extra virgin olive oil intake, the olive oil group consumed on average 38.8 ± 10.5 mL/day and the olive oil + DieTBra group ingested around 37.5 ± 11.69 mL/day, without statistical difference (*p* = 0.634).

Comparing the data from the beginning and the end of the study, the serum calcium levels showed a significant increase in the olive oil group (*p* = 0.007) and PTH showed a significant reduction in the DieTBra group (*p* = 0.047). There was a decrease in energy intake in the DieTBra group (*p* = 0.000) and in protein intake in olive oil (*p* = 0.001) and olive oil + DieTBra (*p* = 0.007) groups (Table 2).

At the end of follow-up, the final BMD mean for the total spine was significantly higher in the DieTBra group in the between-groups comparison (*p* = 0.040). In a paired comparison, the total spine BMD (*p* = 0.016) and total hip BMD (*p* = 0.029) were significantly higher in the DieTBra group than the olive oil + DieTBra group. Final mean serum calcium levels were higher in the olive oil group than in the olive oil + DieTBra group. Energy intake was significantly lower in DieTBra group compared to olive oil group, both in the between-groups comparison (*p* = 0.002) and in the paired comparison of intervention groups (*p* = 0.019). DieTBra group presented the same findings compared to the olive oil + DieTBra group (*p* = 0.031) (Table 3).

Delta value of the outcome variables was compared between the three groups and by pairs. A significant reduction was observed in delta PTH of the DieTBra group in relation to the olive oil + DieTBra group. Delta energy intake was higher in the DieTBra group than the olive oil group, both in the between-groups comparison (*p* = 0.016) and in the paired comparison (*p* = 0.005) (Table 4).

The delta weight was 1.43 ± 1.97 kg for the olive oil group, −1.23 ± 3.18 kg for the DieTBra group, and −1.16 ± 2.8 kg for the olive oil + DieTBra group, with a significant difference (*p* < 0.001). Only the final mean serum calcium and delta serum calcium in the olive oil + DieTBra group met the requirements to perform ANCOVA (*p* = 0.029 and *p* = 0.015). The delta weight influenced the serum calcium results in the olive oil + DieTBra group, but it had no effect on BMD.

## 4. Discussion

To our knowledge, this is the first RCT evaluating the effects of extra virgin olive oil and DieTBra on BMD and levels of serum calcium, vitamin D, and PTH in severely obese participants. There are few RCTs which analyze the effectiveness of dietary interventions on bone health parameters; the majority have only evaluated supplementation of calcium and/or vitamin D [54]. Therefore, this study contributes to the knowledge with important information on bone health and severe obesity and could be a precursor for future research on this topic. Specifically, it demonstrates a significant increase in spine and hip BMD, as well as a significant reduction in PTH levels in participants of the DieTBra group, even with lower energy intake compared to the other groups, and an increase in serum calcium in the olive oil group after a 12-week follow-up.

The significant increase in total spine and total hip BMD in the DieTBra group corroborates findings by Rivas et al. [55], who found that calcaneal BMD in pre- and postmenopausal women was significantly associated with the regular consumption of fruits and vegetables within a healthy and varied diet pattern (Mediterranean diet). Another study involving Mexican adults identified specific dietary patterns associated with BMD, highlighting the importance of promoting strategies to maintain bone health through healthy eating, consistent with the dietary habits of the population [56].

A reduction in PTH was also observed in the DieTBra group after 12 weeks of follow-up. This result is particularly important because the concentration of bone calcium is balanced with the concentration of serum calcium, which is regulated by PTH. Once increases in PTH activate bone remodeling, increasing blood calcium levels, a decrease in PTH may be beneficial, favoring the aggregation of calcium in the bones, whereas this mineral exerts feedback inhibition on the PTH glands as well [57]. In this study, the DieTBra improved levels of this bone marker, suggesting a better balance of PTH levels after a nutritional intervention with a healthy dietary pattern, different from another study with the Dietary Approaches to Stop Hypertension (DASH), in which there was an increase only in calcitriol levels without a difference for PTH and further bone parameters during three weeks of follow-up [58].

A significant increase in calcium was observed in the olive oil group, consistent with the results of the study Prevención con Diet Mediterranea (PREDIMED), involving elderly participants with high cardiovascular risk [59]. The hypolipidic diet group and the MD + chestnuts group demonstrated a significant reduction of serum calcium (*p* = 0.001 and *p* = 0.02), while the MD + extra virgin olive oil group maintained a normal serum calcium concentration [59]. An animal model study observed an increase in BMD after 12 weeks of intervention with EVOO, but no difference for serum calcium [10].

A further study involving pre- and postmenopausal women showed that the absorption of calcium was associated with higher fat intake and lower fiber intake [60]. High fat intake and low dietary fiber intake are commonly found in obese adults [61]. Therefore, in this study, the higher fat content consumed in the olive oil group, with subjects maintaining their usual diet and adding extra virgin olive oil supplementation, may have favored the increase in serum calcium. It is postulated that higher fat content can increase the absorption of calcium due to the lipid solubility of vitamin D, which favors the absorption of 30% to 40% of intestinal calcium [62]. Interestingly, we observed a slight increase in vitamin D levels in the olive oil and DieTBra groups in our study, and although not statistically significant, these findings are clinically relevant. In the olive oil group, a reduction in protein intake was also observed at the end of the study. However, the amount of protein ingested still remained within the DRIs. We believe that this small reduction in protein intake will not affect BMD due to the positive effects of olive oil. A recent study showed that elderly participants in the highest tertile of EVOO consumption had a 51% lower risk of fracture [14].

There was no statistically significant effect on the primary outcomes with concomitant use of EVOO and DieTBra in severely obese individuals, even though EVOO is composed of polyphenols and tocopherols, substances that have antioxidant and anti-inflammatory properties [13,63]. Our hypothesis is related to the nutritional prescription for this group (olive oil + DieTBra), since, with EVOO supplementation, there was a reduction of 468 kcal in food for isocaloric equivalence of the nutritional prescription of the DieTBra group. With a reduction in the amount of food, adherence to treatment is more difficult, especially considering that they were adults with severe obesity accustomed to eating large amounts of food. In general, obese people have low adherence to nutritional therapies for several reasons [64], such as quantitative food restrictions and the possible difficulty of following two concomitant nutritional interventions.

It is important to highlight the dietary intervention used on this study, DieTBra, was consistent with regional dietary habits. Future studies should consider the food consumption pattern before defining which nutritional intervention will be assigned to the target population of a study. The Mediterranean diet is a complicated diet pattern to be followed by other populations in countries located in large areas such as Latin America and Asia, whose eating habits are very different from the Mediterranean regions. Furthermore, this study highlights the benefits of a balanced diet, characterized by an adequate consumption of foods, such as rice, beans, fruits and vegetables, dairy products, and low ingestion of ultra-processed foods [23,65], which can improve overall health, including bone health.

It is interesting to discuss that the average daily consumption of extra virgin olive oil was 38.8 ± 10.5 mL in olive oil and 37.5 ± 11.69 mL in olive oil + DieTBra groups, below the 52 mL initially delivered on the sachets. We should consider it as an effective study, where the participants’ real-life affects the use and adherence of behavioral interventions, such as diet, supplements, and medications [66]. Clinical studies with dietary interventions depend on adherence, actual food consumption, and the food report of the participant that is not under control in a laboratory [67]. It is worth mentioning that although olive oil is very healthy, there is still no high consumption of this oil in Brazil [68] as in the Mediterranean countries [69]. With greater access to information, olive oil has gained more space in Brazilian culture, another point that we consider positive in our study [68].

This study has some limitations. The duration of supplementation with extra virgin olive oil was 12 weeks, a relatively short period of time to verify changes in BMD. Nevertheless, significant effects on BMD, PTH levels, and serum calcium levels were observed during this period. It is important to emphasize the use of those nutritional interventions, like olive oil and DieTBra, that are inexpensive, accessible, and without side effects. One of the strengths of this study is its nutritional approach, the prescription of the DieTBra, which is based on natural and minimally processed foods, in addition to the supplementation with extra virgin olive oil in food form and not in capsules, as a healthy diet is based on food intake and not on tablets or capsules. A further strength is the low loss to follow-up (12.6%), as the original prediction was 25% of the initial study population; generally, obese participants have poor treatment compliance treatment [64]. Furthermore, in future studies with intervention time similar to this research, it is interesting to use protein turnover markers to complement the data obtained.

The results found here increase the knowledge in the area of treatments related to BMD and other bone health parameters in severely obese adults. These results may assist in the development of treatment goals for severely obese adults aimed at preserving bone health and avoiding the adverse effects of BMD reduction. The nutritional interventions used in this study are relatively easy to incorporate into clinical treatments, and because of the absence of side effects, can be widely employed.

## 5. Conclusions

The interventions with DieTBra and extra virgin olive oil over 12 weeks of follow-up had positive effects on bone health in severely obese adults. DieTBra was effective to increase BMD in two sites, total spine and hip, and to decrease PTH levels, while extra virgin olive oil was effective in increasing serum calcium. It is important to emphasize the need for long-term studies to examine whether the combination of extra virgin olive oil and healthy dietary patterns, such as DieTBra, may cause more significant effects on BMD at specific sites and on other markers of bone synthesis in severely obese participants.

## Figures and Tables

**Table 1:** Characterization of severely obese adults at baseline for the intervention groups, Brazil, 2016 (*n* = 111).

Variables	Total *n* (%)	Olive Oil (*n* = 34) *n* (%)	DieTBra (*n* = 38) *n* (%)	Olive Oil + DieTBra (*n* = 39) *n* (%)
Sex *				
Women	104 (93.7)	32 (94.1)	36 (94.7)	36 (92.3)
Men	7 (6.3)	2 (5.9)	2 (5.3)	3 (7.7)
Age (years) **				
18–40	57 (51.4)	19 (55.9)	21 (55.3)	17 (43.6)
≥41	54 (48.6)	15 (44.1)	17 (44.7)	22 (56.4)
Skin color **				
White/dark	94 (84.7)	29 (85.3)	33 (86.8)	32 (82.0)
Black	17 (15.3)	5 (14.7)	5 (13.2)	7 (18.0)
Formal education (years of study) **				
≤4	10 (9.0)	1 (2.9)	2 (5.3)	7 (17.9)
5–11	83 (74.8)	27 (79.4)	30(78.9)	26 (66.7)
≥12	18 (16.2)	6 (17.7)	6(15.8)	6 (15.4)
Economic class **				
A-B	28 (25.2)	12 (35.3)	7 (18.4)	9 (23.1)
C	65 (58.6)	15 (44.1)	24 (63.2)	26 (66.7)
D-E	18 (16.2)	7 (20.6)	7 (18.4)	4(10.2)
Smoking**				
Never smoked	76 (68.5)	25 (73.5)	27 (71.0)	24 (61.5)
Smoker/ex-smoker	35 (31.5)	9 (26.5)	11 (29.0)	15 (38.5)
Binge drinking (*n* = 62) **				
Yes	32 (51.6)	9 (50.0)	10 (47.6)	13 (56.5)
No	30 (48.4)	9 (50.0)	11 (52.4)	10 (43.5)
Menopause (*n* = 104) *				
Yes	18 (16.2)	6 (18.7)	3 (8.3)	9 (25.0)
No	86 (77.5)	26 (81.3)	33 (91.7)	27 (75.0)
Drugs↓BMD **				
Yes	28 (25.2)	6 (17.6)	8 (21.1)	14 (35.9)
No	83 (74.8)	28 (82.4)	30 (78.9)	25 (64.1)
PA ≥150 min/week (*n* = 104) *				
Yes	99 (95.2)	30 (93.8)	35 (97.2)	34 (94.5)
No	4 (4.8)	2 (6.2)	1 (2.8)	2 (5.6)
BMI (kg/m^2^) *				
35–39.99	22 (19.8)	7 (20.6)	8 (21.1)	7 (18.0)
40–49.99	78 (70.3)	25 (73.5)	23 (60.5)	30 (76.9)
>50	11(9.9)	2 (5.9)	7 (18.4)	2 (5.1)
Weight (mean kg ± SD) ***	110.211.6	108.0 ± 10.9	111.7 ± 12.1	110.7 ± 11.8
Fat mass (mean kg ± SD) ***	51.6 ± 5.2	50.5 ± 5.1	51.5 ± 4.7	52.6 ± 5.6
Lean mass (mean kg ± SD)	50.7 ± 9.1	47.9 ± 11.1	51.9 ± 7.6	50.4 ± 8.5
**Outcomes**	**Mean ± SD**	**Mean ± SD**	**Mean ± SD**	**Mean ± SD**
BMD spine (g/cm^2^) ***	1.069 ± 0.165	1.053 ± 0.136	1.119 ± 0.166	1.035 ± 0.180
BMD hip (g/cm^2^) ***	1.201 ± 0.143	1.182 ± 0.139	1.236 ± 0.120	1.183 ± 0.162
Serum calcium (mg/dL) ***	9.5 ± 0.5	9.4 ± 0.5	9.6 ± 0.6	9.4 ± 0.41
Serum vitamin D (ng/mL) ***	30.5 ± 9.6	30.8 ± 9.5	29.16 ± 9.2	31.5 ± 10.1
PTH (pg/mL) ***	53.7 ± 26.0	55.0 ± 20.7	57.9 ± 35.9	48.5 ± 16.5
Dietary calcium (mg/day) ***	417.2 ± 222.6	375.5 ± 213.0	445.0 ± 210.9	426.5 ± 241.5
Dietary vitamin D (µcg/day) ***	2.1 ± 1.8	1.8 ± 1.6	2.4 ± 1.6	2.1 ± 2.2
Energy intake (kcal/day) ****	1685.1 ± 762.1	1674.8 ± 610.8	1678.1 ± 568.2	1701.1 ± 1021.1
Protein intake (% TEV) ***	18.0 ± 4.8	18.1 ± 4.3	17.3 ± 5.1	18.7 ± 4.7

* Fisher’s exact test (*p* > 0.05). ** Chi-squared test (*p* > 0.05). *** ANOVA (*p* > 0.05). **** Kruskal–Wallis (*p* > 0.05). ↓(reduces). DieTBra (traditional Brazilian diet), BMD (bone mineral density), PA (physical activity), SD (standard deviation), BMI (body mass index), PTH (parathyroid hormone), TEV (total energy value).

**Table 2:** Baseline and outcome variables in all three intervention groups with severe obesity, Brazil, 2016 (*n* = 111).

	Olive Oil			DieTBra			Olive Oil + DieTBra		
	Mean ± SD			Mean ± SD			Mean ± SD		
Outcomes	Before (*n* = 34)	After (*n* = 28)	*p* *	Before (*n* = 38)	After (*n* = 32)	*p* *	Before (*n* = 39)	After (*n* = 37)	*p* *
BMD spine total (g/cm^2^)	1.053 ± 0.136	1.092 ± 0.139	0.269	1.119 ± 0.166	1.145 ± 0.157	0.512	1.035 ± 0.179	1.044 ± 0.177	0.806
BMD hip total (g/cm^2^)	1.183 ± 0.139	1.214 ± 0.125	0.364	1.236 ± 0.119	1.244 ± 0.151	0.792	1.183 ± 0.162	1.164 ± 0.149	0.588
Serum calcium (mg/dL)	9.5 ± 0.5	9.8 ± 0.5	**0.007**	9.5 ± 0.5	9.6 ± 0.6	0.622	9.4 ± 0.4	9.5 ± 0.5	0.330
Vit. D serum (ng/mL)	30.8 ± 9.5	31.3 ± 9.8	0.823	29.2 ± 9.2	31.7 ± 9.7	0.255	31.5 ± 10.1	31.5 ± 9.9	0.998
PTH (pg/ml)	55.0 ± 20.7	45.9 ± 16.4	0.065	57.9 ± 35.9	44.6 ± 13.6	**0.047**	48.5 ± 16.5	47.5 ± 15.2	0.791
Dietary calcium (mg/day)	375.5 ± 213.0	362.4 ± 257.4	0.819	445.0 ± 210.9	341.1 ± 263.6	**0.061**	426.5 ± 241.5	358.0 ± 238.2	0.211
Dietary vitamin D (µcg/day)	1.8 ± 1.4	1.9 ± 2.1	0.710	2.6 ± 1.9	3.3 ± 3.6	0.214	2.1 ± 2.0	1.9 ± 1.9	0.773
Energy intake (kcal/day)	1674.8 ± 610.8	1529.9 ± 885.0	0.434	1678.1 ± 568.2	1104.1 ± 609.7	**0.000**	1701.1 ± 1021.1	1397.3 ± 567.0	0.108
Protein intake (%TEV)	18.1 ± 4.3	13.2 ± 7.1	**0.001**	17.3 ± 5.1	15.9 ± 8.6	0.374	18.7 ± 4.7	14.9 ± 7.2	**0.007**

BMD (bone mineral density), Vit. D (vitamin D), PTH (parathyroid hormone), SD (standard deviation), olive oil (extra virgin olive oil group), DieTBra (traditional Brazilian diet group), olive oil + DieTBra (extra virgin olive oil group + traditional Brazilian diet), TEV (total energy value). * Student’s t test unpaired. Bold (value *p* < 0.05).

**Table 3:** Outcome variables at the end of follow-up in the three intervention groups with severe obesity, Brazil, 2016 (*n* = 97).

	Olive Oil (*n* = 28)	DieTBra (*n* = 32)	Olive Oil + DieTBra (*n* = 37)	All	Olive Oil × DieTBra	Olive Oil × Olive Oil + DieTBra	DieTBra × Olive Oil + DieTBra
Outcomes	Mean ± SD	Mean ± SD	Mean ± SD	*p*	*p*	*p*	*p*
BMD spine total (g/cm^2^)	1.092 ± 0.139	1.145 ± 0.157 ^a^	1.044 ± 0.177 ^a^	0.040 *	0.178 ***	0.246 ***	0.016 ***
BMD hip total (g/cm^2^)	1.214 ± 0.125	1.244 ± 0.151	1.164 ± 0.149	0.066 *	0.397 ***	0.157 ***	0.029 ***
Serum calcium (mg/dL)	9.8 ± 0.5	9.6 ± 0.6	9.5 ± 0.5	0.092 *	0.137 ***	0.026 ***	0.535 ***
Vit. D serum (ng/mL)	31.3 ± 9.8	31.7 ± 9.7	31.5 ± 9.9	0.988 *	0.879 ***	0.947 ***	0.926 ***
PTH (pg/mL)	45.9 ± 16.4	44.6 ± 13.6	47.5 ± 15.2	0.721 *	0.710 ***	0.688 ***	0.404 ***
Dietary calcium (mg/day)	362.4 ± 257.4	341.1 ± 263.6	358.0 ± 238.2	0.930 *	0.731 ***	0.940 ***	0.768 ***
Dietary vitamin D (µcg/day)	1.9 ± 2.1	3.3 ± 3.6	1.9 ± 1.9	0.257 **	0.220 ****	0.712 ****	0.124 ****
Energy intake (kcal/day)	1529.9 ± 885.0 ^a^	1104.1 ± 609.7 ^a,b^	1397.3 ± 567.0 ^b^	**0.002** **	**0.019** ***	0.443 ***	**0.031** ***
Protein intake (%TEV)	13.2 ± 7.1	15.9 ± 8.6	14.9 ± 7.2	0.336 *	0.157 ***	0.315 ***	0.593 ***

BMD (bone mineral density), Vit. D (vitamin D), PTH (parathyroid hormone), SD (standard deviation), TEV (total energy value), all (comparison between the three groups), olive oil (extra virgin olive oil group), DieTBra (traditional Brazilian diet group), olive oil + DieTBra (extra virgin olive oil group + traditional Brazilian diet). * ANOVA. ** Kruskal–Wallis (energy–normal distribution, but without homoscedasticity; dietary vitamin D with non-normal distribution). *** Student’s t-test. **** Mann–Whitney. Means followed by the same letter are statistically different from each other. Bold (value *p* < 0.05).

**Table 4:** Difference between post and pre-intervention of the outcome variables in the three intervention groups with severe obesity, Brazil, 2016 (*n* = 97).

	Olive Oil (*n* = 28)	DieTBra (*n* = 32)	Olive Oil + DieTBra (*n* = 37)	All *	Olive Oil × DieTBra **	Olive Oil × Olive Oil + DieTBra **	DieTBra × Olive Oil + DieTBra **
Outcomes (Final-Initial)	Mean ± SD	Mean ± SD	Mean ± SD	*p*	*p*	*p*	*p*
▲BMD spine (g/cm^2^)	0.034 ± 0.116	0.043 ± 0.092	0.009 ± 0.111	0.399 *	0.754	0.380	0.180
▲BMD hip (g/cm^2^)	0.026 ± 0.111	0.009 ± 0.100	−0.003 ± 0.109	0.556 *	0.531	0.296	0.641
▲Serum calcium (mg/dL)	0.4 ± 0.7	−0.0 ± 0.8	0.1 ± 0.7	0.116 *	0.051	0.122	0.536
▲ Serum Vit. D (ng/mL)	0.1 ± 6.5	2.7 ± 8.4	0.7 ± 5.4	0.675 **	0.218	0.697	0.273
▲PTH (pg/mL)	−7.3 ± 20.7	−12.6 ± 29.9 ^a^	−1.2 ± 13.2 ^a^	0.10 **	0.431	0.158	0.042
▲Dietary calcium (mg/day)	−13.1 ± 294.8	−103.8 ± 268.3	−68.4 ± 218.4	0.337 *	0.176 ***	0.362 ***	0.526 ***
▲Dietary vitamin D (µcg/day)	0.2 ± 2.9	0.9 ± 3.9	−0.2 ± 2.4	0.831 **	0.458 ***	0.448 ***	0.129 ***
▲Energy intake (kcal/day)	−144.8 ± 918.3 ^a^	−573.9 ± 719.8 ^a^	−303.7 ± 882.1	**0.016** **	**0.005** ****	0.325 ****	0.055 ****
▲Protein intake (%TEV)	−4.9 ± 7.1	−1.4 ± 10.2	−3.8 ± 8.3	0.227 *	0.105 ***	0.560 ***	0.265 ***

▲ (difference), BMD (bone mineral density), Vit. D (vitamin D), PTH (parathyroid hormone), SD (standard deviation), TEV (total energy value), all (comparison between the three groups), olive oil (extra virgin olive oil group), DieTBra (traditional Brazilian diet group), olive oil + DieTBra (extra virgin olive oil group + traditional Brazilian diet). * ANOVA. ** Kruskal–Wallis test for delta serum vitamin D, PTH, dietary vitamin D (normal distribution, but without homoscedasticity; energy with non-normal distribution). *** Student’s t-test. **** Man–Whitney. Means followed by the same letter are statistically different from each other. Bold (value *p* < 0.05).
